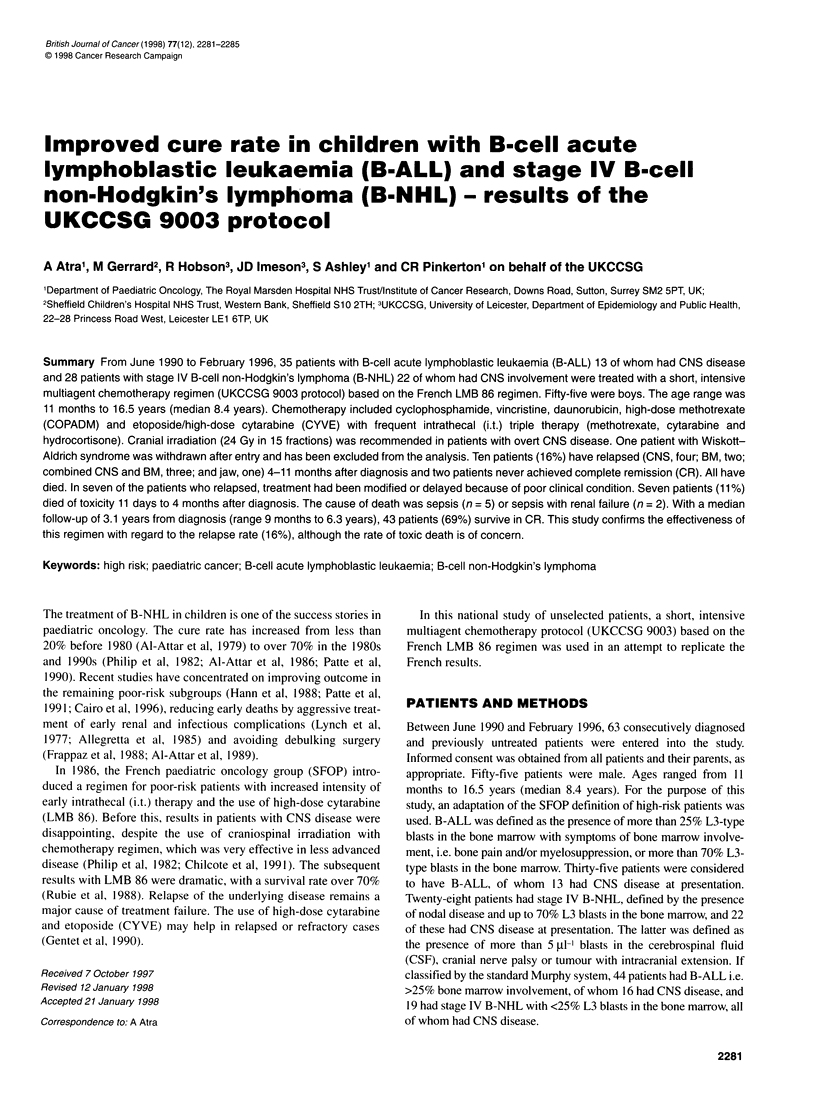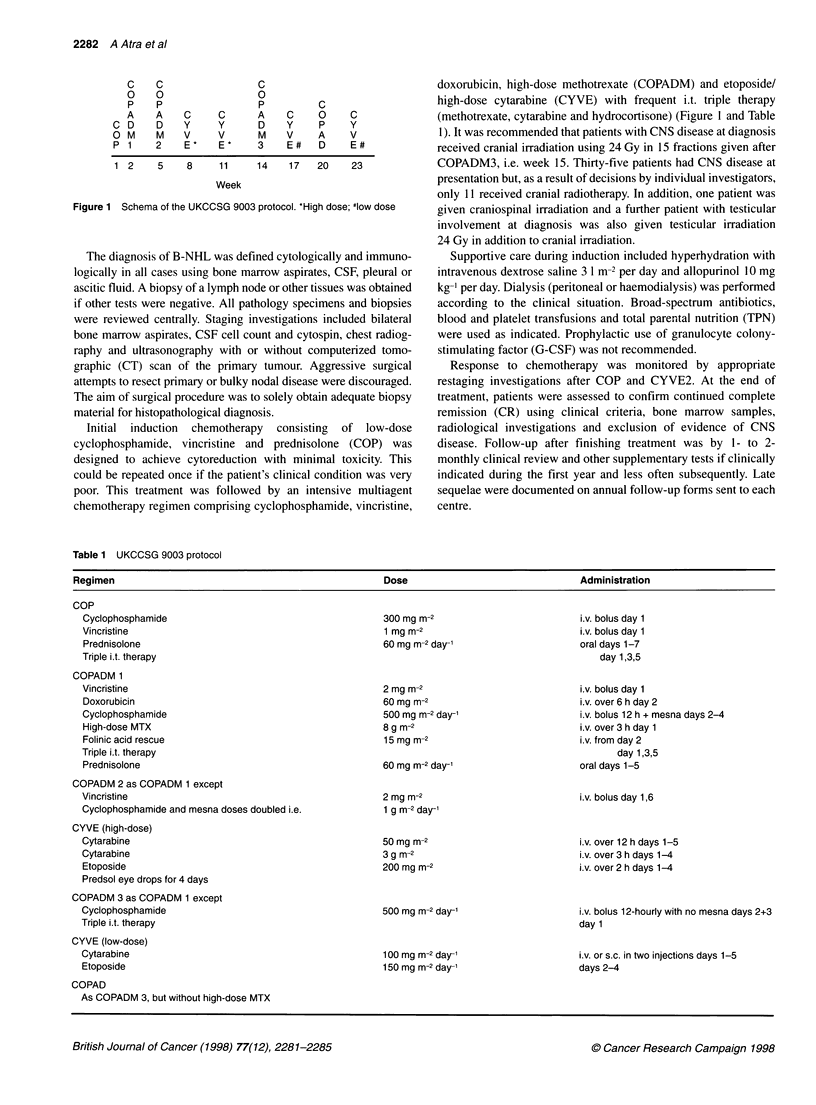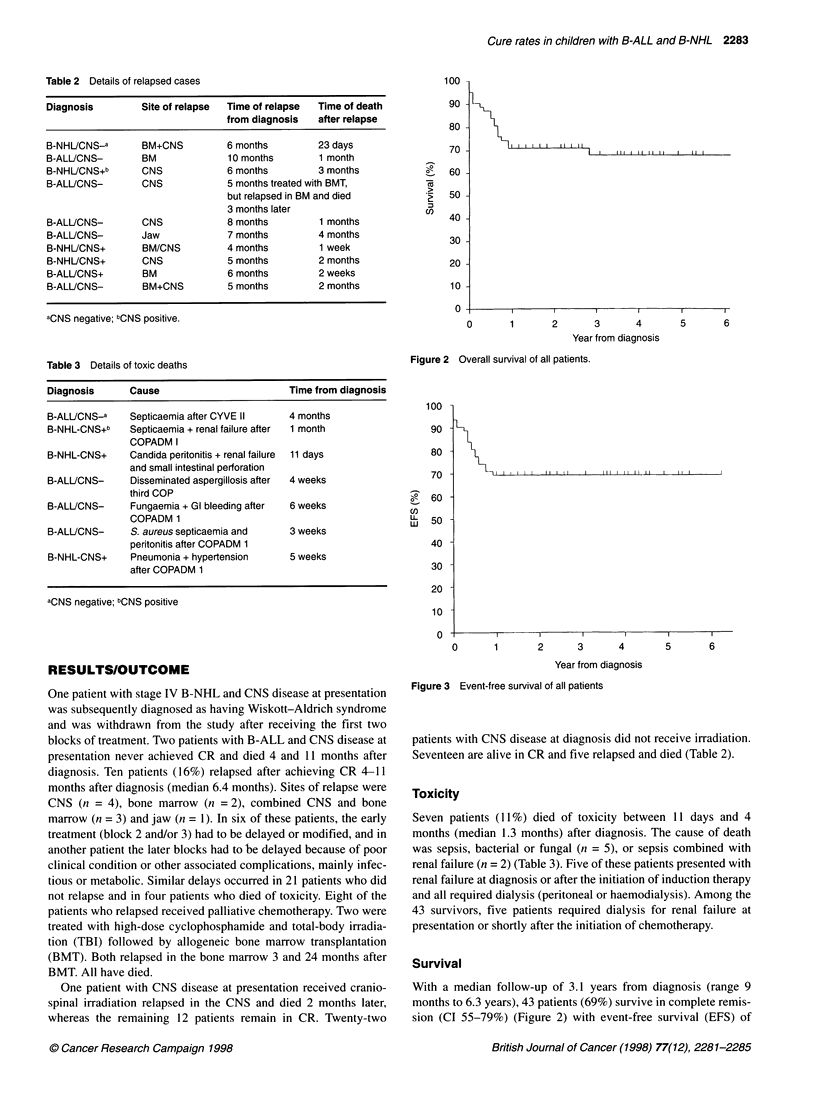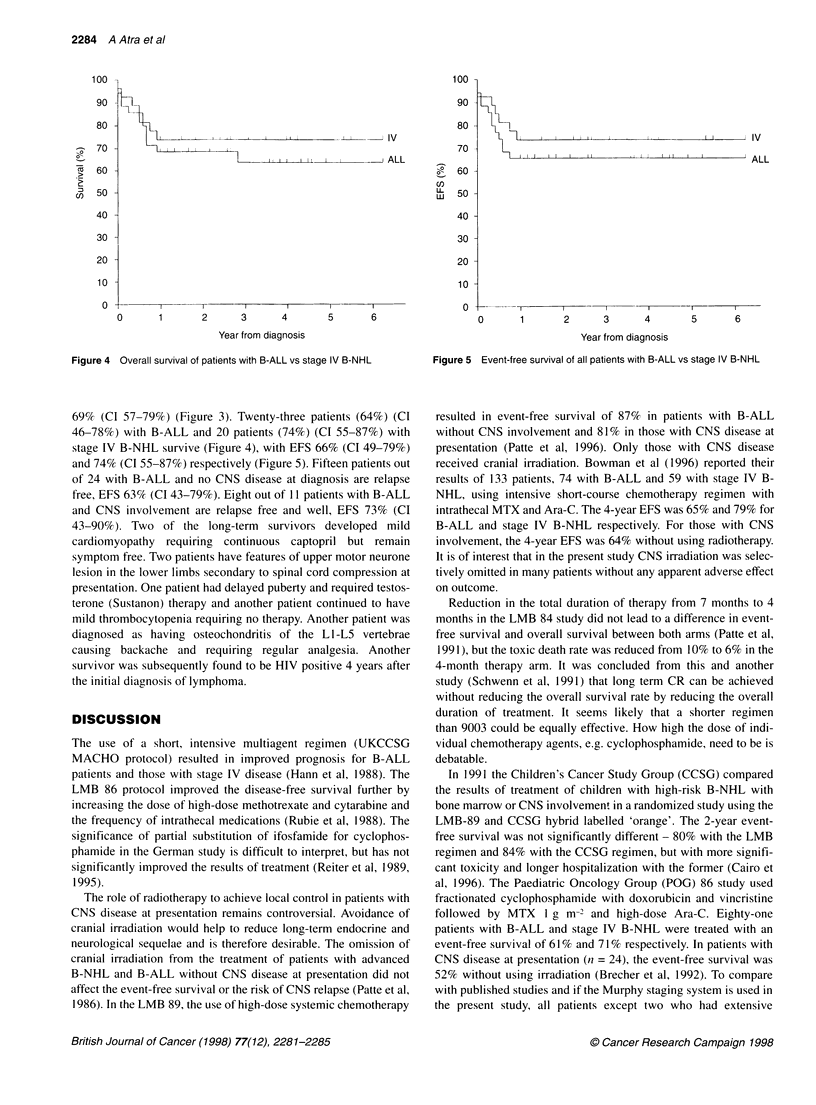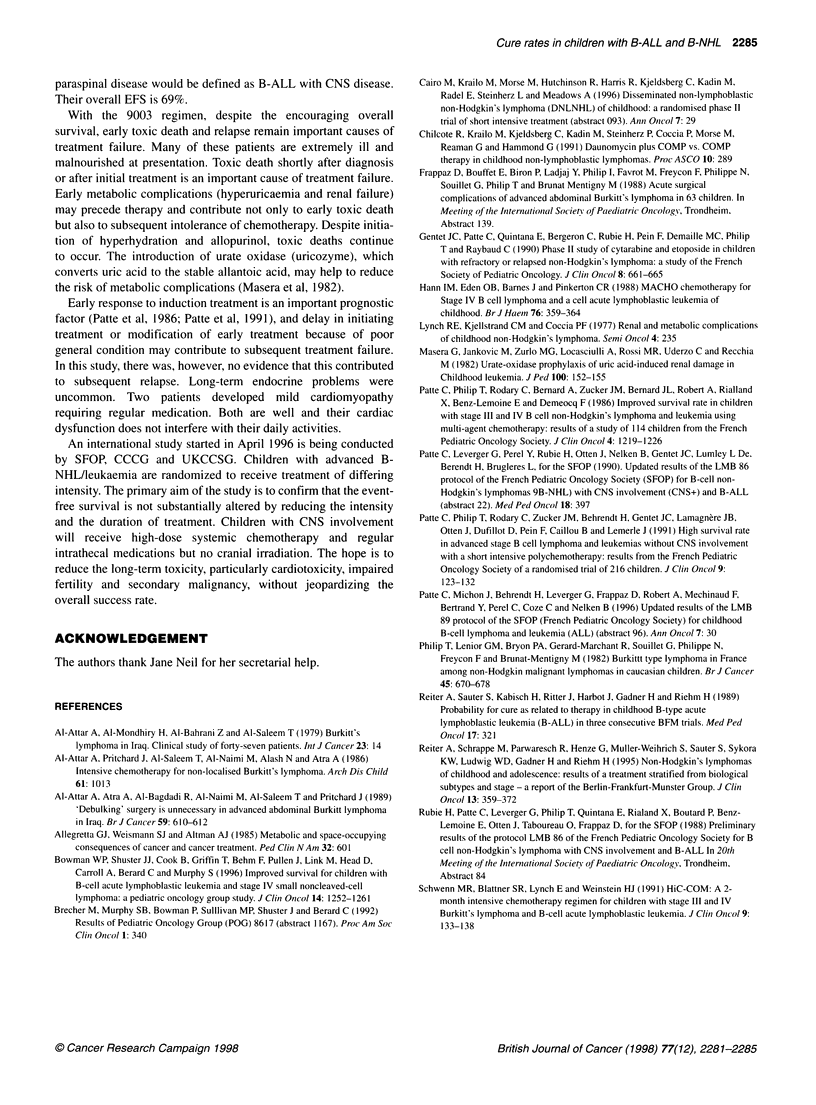# Improved cure rate in children with B-cell acute lymphoblastic leukaemia (B-ALL) and stage IV B-cell non-Hodgkin's lymphoma (B-NHL)--results of the UKCCSG 9003 protocol.

**DOI:** 10.1038/bjc.1998.379

**Published:** 1998-06

**Authors:** A. Atra, M. Gerrard, R. Hobson, J. D. Imeson, S. Ashley, C. R. Pinkerton

**Affiliations:** Department of Paediatric Oncology, The Royal Marsden Hospital NHS Trust/Institute of Cancer Research, Sutton, Surrey, UK.

## Abstract

From June 1990 to February 1996, 35 patients with B-cell acute lymphoblastic leukaemia (B-ALL) 13 of whom had CNS disease and 28 patients with stage IV B-cell non-Hodgkin's lymphoma (B-NHL) 22 of whom had CNS involvement were treated with a short, intensive multiagent chemotherapy regimen (UKCCSG 9003 protocol) based on the French LMB 86 regimen. Fifty-five were boys. The age range was 11 months to 16.5 years (median 8.4 years). Chemotherapy included cyclophosphamide, vincristine, daunorubicin, high-dose methotrexate (COPADM) and etoposide/high-dose cytarabine (CYVE) with frequent intrathecal (i.t.) triple therapy (methotrexate, cytarabine and hydrocortisone). Cranial irradiation (24 Gy in 15 fractions) was recommended in patients with overt CNS disease. One patient with Wiskott-Aldrich syndrome was withdrawn after entry and has been excluded from the analysis. Ten patients (16%) have relapsed (CNS, four; BM, two; combined CNS and BM, three; and jaw, one) 4-11 months after diagnosis and two patients never achieved complete remission (CR). All have died. In seven of the patients who relapsed, treatment had been modified or delayed because of poor clinical condition. Seven patients (11%) died of toxicity 11 days to 4 months after diagnosis. The cause of death was sepsis (n = 5) or sepsis with renal failure (n = 2). With a median follow-up of 3.1 years from diagnosis (range 9 months to 6.3 years), 43 patients (69%) survive in CR. This study confirms the effectiveness of this regimen with regard to the relapse rate (16%), although the rate of toxic death is of concern.


British Journal of Cancer (1998) 77(12), 2281–2285
© 1998 Cancer Research Campaign

### Improved cure rate in children with B-cell acute lymphoblastic leukaemia (B-ALL) and stage IV B-cell non-Hodgkin's lymphoma (B-NHL) – results of the UKCCSG 9003 protocol

**A Atra[1], M Gerrard[2], R Hobson[3], JD Imeson[3], S Ashley[1] and CR Pinkerton[1] on behalf of the UKCCSG**

[1]Department of Paediatric Oncology, The Royal Marsden Hospital NHS Trust/Institute of Cancer Research, Downs Road, Sutton, Surrey SM2 5PT, UK; [2]Sheffield Children's Hospital NHS Trust, Western Bank, Sheffield S10 2TH; [3]UKCCSG, University of Leicester, Department of Epidemiology and Public Health, 22–28 Princess Road West, Leicester LE1 6TP, UK

**Summary** From June 1990 to February 1996, 35 patients with B-cell acute lymphoblastic leukaemia (B-ALL) 13 of whom had CNS disease and 28 patients with stage IV B-cell non-Hodgkin's lymphoma (B-NHL) 22 of whom had CNS involvement were treated with a short, intensive multiagent chemotherapy regimen (UKCCSG 9003 protocol) based on the French LMB 86 regimen. Fifty-five were boys. The age range was 11 months to 16.5 years (median 8.4 years). Chemotherapy included cyclophosphamide, vincristine, daunorubicin, high-dose methotrexate (COPADM) and etoposide/high-dose cytarabine (CYVE) with frequent intrathecal (i.t.) triple therapy (methotrexate, cytarabine and hydrocortisone). Cranial irradiation (24 Gy in 15 fractions) was recommended in patients with overt CNS disease. One patient with Wiskott–Aldrich syndrome was withdrawn after entry and has been excluded from the analysis. Ten patients (16%) have relapsed (CNS, four; BM, two; combined CNS and BM, three; and jaw, one) 4–11 months after diagnosis and two patients never achieved complete remission (CR). All have died. In seven of the patients who relapsed, treatment had been modified or delayed because of poor clinical condition. Seven patients (11%) died of toxicity 11 days to 4 months after diagnosis. The cause of death was sepsis ($n = 5$) or sepsis with renal failure ($n = 2$). With a median follow-up of 3.1 years from diagnosis (range 9 months to 6.3 years), 43 patients (69%) survive in CR. This study confirms the effectiveness of this regimen with regard to the relapse rate (16%), although the rate of toxic death is of concern.

**Keywords:** high risk; paediatric cancer; B-cell acute lymphoblastic leukaemia; B-cell non-Hodgkin's lymphoma

The treatment of B-NHL in children is one of the success stories in paediatric oncology. The cure rate has increased from less than 20% before 1980 (Al-Attar et al, 1979) to over 70% in the 1980s and 1990s (Philip et al, 1982; Al-Attar et al, 1986; Patte et al, 1990). Recent studies have concentrated on improving outcome in the remaining poor-risk subgroups (Hann et al, 1988; Patte et al, 1991; Cairo et al, 1996), reducing early deaths by aggressive treatment of early renal and infectious complications (Lynch et al, 1977; Allegretta et al, 1985) and avoiding debulking surgery (Frappaz et al, 1988; Al-Attar et al, 1989).

In 1986, the French paediatric oncology group (SFOP) introduced a regimen for poor-risk patients with increased intensity of early intrathecal (i.t.) therapy and the use of high-dose cytarabine (LMB 86). Before this, results in patients with CNS disease were disappointing, despite the use of craniospinal irradiation with chemotherapy regimen, which was very effective in less advanced disease (Philip et al, 1982; Chilcote et al, 1991). The subsequent results with LMB 86 were dramatic, with a survival rate over 70% (Rubie et al, 1988). Relapse of the underlying disease remains a major cause of treatment failure. The use of high-dose cytarabine and etoposide (CYVE) may help in relapsed or refractory cases (Gentet et al, 1990).

*Received 7 October 1997*
*Revised 12 January 1998*
*Accepted 21 January 1998*

*Correspondence to:* A Atra

In this national study of unselected patients, a short, intensive multiagent chemotherapy protocol (UKCCSG 9003) based on the French LMB 86 regimen was used in an attempt to replicate the French results.

### PATIENTS AND METHODS

Between June 1990 and February 1996, 63 consecutively diagnosed and previously untreated patients were entered into the study. Informed consent was obtained from all patients and their parents, as appropriate. Fifty-five patients were male. Ages ranged from 11 months to 16.5 years (median 8.4 years). For the purpose of this study, an adaptation of the SFOP definition of high-risk patients was used. B-ALL was defined as the presence of more than 25% L3-type blasts in the bone marrow with symptoms of bone marrow involvement, i.e. bone pain and/or myelosuppression, or more than 70% L3-type blasts in the bone marrow. Thirty-five patients were considered to have B-ALL, of whom 13 had CNS disease at presentation. Twenty-eight patients had stage IV B-NHL, defined by the presence of nodal disease and up to 70% L3 blasts in the bone marrow, and 22 of these had CNS disease at presentation. The latter was defined as the presence of more than 5 $\mu l^{-1}$ blasts in the cerebrospinal fluid (CSF), cranial nerve palsy or tumour with intracranial extension. If classified by the standard Murphy system, 44 patients had B-ALL i.e. >25% bone marrow involvement, of whom 16 had CNS disease, and 19 had stage IV B-NHL with <25% L3 blasts in the bone marrow, all of whom had CNS disease.

| Week | 1 | 2 | 5 | 8 | 11 | 14 | 17 | 20 | 23 |
|---|---|---|---|---|---|---|---|---|---|
| Regimen | COP | COPADM 1 | COPADM 2 | CYVE* | CYVE* | COPADM 3 | CYVE# | COPAD | CYVE# |

**Figure 1** Schema of the UKCCSG 9003 protocol. *High dose; #low dose

The diagnosis of B-NHL was defined cytologically and immunologically in all cases using bone marrow aspirates, CSF, pleural or ascitic fluid. A biopsy of a lymph node or other tissues was obtained if other tests were negative. All pathology specimens and biopsies were reviewed centrally. Staging investigations included bilateral bone marrow aspirates, CSF cell count and cytospin, chest radiography and ultrasonography with or without computerized tomographic (CT) scan of the primary tumour. Aggressive surgical attempts to resect primary or bulky nodal disease were discouraged. The aim of surgical procedure was to solely obtain adequate biopsy material for histopathological diagnosis.

Initial induction chemotherapy consisting of low-dose cyclophosphamide, vincristine and prednisolone (COP) was designed to achieve cytoreduction with minimal toxicity. This could be repeated once if the patient's clinical condition was very poor. This treatment was followed by an intensive multiagent chemotherapy regimen comprising cyclophosphamide, vincristine, doxorubicin, high-dose methotrexate (COPADM) and etoposide/high-dose cytarabine (CYVE) with frequent i.t. triple therapy (methotrexate, cytarabine and hydrocortisone) (Figure 1 and Table 1). It was recommended that patients with CNS disease at diagnosis received cranial irradiation using 24 Gy in 15 fractions given after COPADM3, i.e. week 15. Thirty-five patients had CNS disease at presentation but, as a result of decisions by individual investigators, only 11 received cranial radiotherapy. In addition, one patient was given craniospinal irradiation and a further patient with testicular involvement at diagnosis was also given testicular irradiation 24 Gy in addition to cranial irradiation.

Supportive care during induction included hyperhydration with intravenous dextrose saline 3 l $m^{-2}$ per day and allopurinol 10 mg $kg^{-1}$ per day. Dialysis (peritoneal or haemodialysis) was performed according to the clinical situation. Broad-spectrum antibiotics, blood and platelet transfusions and total parental nutrition (TPN) were used as indicated. Prophylactic use of granulocyte colony-stimulating factor (G-CSF) was not recommended.

Response to chemotherapy was monitored by appropriate restaging investigations after COP and CYVE2. At the end of treatment, patients were assessed to confirm continued complete remission (CR) using clinical criteria, bone marrow samples, radiological investigations and exclusion of evidence of CNS disease. Follow-up after finishing treatment was by 1- to 2-monthly clinical review and other supplementary tests if clinically indicated during the first year and less often subsequently. Late sequelae were documented on annual follow-up forms sent to each centre.

**Table 1** UKCCSG 9003 protocol

| Regimen | Dose | Administration |
|---|---|---|
| COP | | |
| Cyclophosphamide | 300 mg $m^{-2}$ | i.v. bolus day 1 |
| Vincristine | 1 mg $m^{-2}$ | i.v. bolus day 1 |
| Prednisolone | 60 mg $m^{-2}$ $day^{-1}$ | oral days 1–7 |
| Triple i.t. therapy | | day 1,3,5 |
| COPADM 1 | | |
| Vincristine | 2 mg $m^{-2}$ | i.v. bolus day 1 |
| Doxorubicin | 60 mg $m^{-2}$ | i.v. over 6 h day 2 |
| Cyclophosphamide | 500 mg $m^{-2}$ $day^{-1}$ | i.v. bolus 12 h + mesna days 2–4 |
| High-dose MTX | 8 g $m^{-2}$ | i.v. over 3 h day 1 |
| Folinic acid rescue | 15 mg $m^{-2}$ | i.v. from day 2 |
| Triple i.t. therapy | | day 1,3,5 |
| Prednisolone | 60 mg $m^{-2}$ $day^{-1}$ | oral days 1–5 |
| COPADM 2 as COPADM 1 except | | |
| Vincristine | 2 mg $m^{-2}$ | i.v. bolus day 1,6 |
| Cyclophosphamide and mesna doses doubled i.e. | 1 g $m^{-2}$ $day^{-1}$ | |
| CYVE (high-dose) | | |
| Cytarabine | 50 mg $m^{-2}$ | i.v. over 12 h days 1–5 |
| Cytarabine | 3 g $m^{-2}$ | i.v. over 3 h days 1–4 |
| Etoposide | 200 mg $m^{-2}$ | i.v. over 2 h days 1–4 |
| Predsol eye drops for 4 days | | |
| COPADM 3 as COPADM 1 except | | |
| Cyclophosphamide | 500 mg $m^{-2}$ $day^{-1}$ | i.v. bolus 12-hourly with no mesna days 2+3 |
| Triple i.t. therapy | | day 1 |
| CYVE (low-dose) | | |
| Cytarabine | 100 mg $m^{-2}$ $day^{-1}$ | i.v. or s.c. in two injections days 1–5 |
| Etoposide | 150 mg $m^{-2}$ $day^{-1}$ | days 2–4 |
| COPAD | | |
| As COPADM 3, but without high-dose MTX | | |

© Cancer Research Campaign 1998

**Table 2** Details of relapsed cases

| Diagnosis | Site of relapse | Time of relapse from diagnosis | Time of death after relapse |
|---|---|---|---|
| B-NHL/CNS–[a] | BM+CNS | 6 months | 23 days |
| B-ALL/CNS– | BM | 10 months | 1 month |
| B-NHL/CNS+[b] | CNS | 6 months | 3 months |
| B-ALL/CNS– | CNS | 5 months treated with BMT, but relapsed in BM and died 3 months later | |
| B-ALL/CNS– | CNS | 8 months | 1 months |
| B-ALL/CNS– | Jaw | 7 months | 4 months |
| B-NHL/CNS+ | BM/CNS | 4 months | 1 week |
| B-NHL/CNS+ | CNS | 5 months | 2 months |
| B-ALL/CNS+ | BM | 6 months | 2 weeks |
| B-ALL/CNS– | BM+CNS | 5 months | 2 months |

[a]CNS negative; [b]CNS positive.

**Table 3** Details of toxic deaths

| Diagnosis | Cause | Time from diagnosis |
|---|---|---|
| B-ALL/CNS–[a] | Septicaemia after CYVE II | 4 months |
| B-NHL-CNS+[b] | Septicaemia + renal failure after COPADM I | 1 month |
| B-NHL-CNS+ | Candida peritonitis + renal failure and small intestinal perforation | 11 days |
| B-ALL/CNS– | Disseminated aspergillosis after third COP | 4 weeks |
| B-ALL/CNS– | Fungaemia + GI bleeding after COPADM 1 | 6 weeks |
| B-ALL/CNS– | *S. aureus* septicaemia and peritonitis after COPADM 1 | 3 weeks |
| B-NHL-CNS+ | Pneumonia + hypertension after COPADM 1 | 5 weeks |

[a]CNS negative; [b]CNS positive

### RESULTS/OUTCOME

One patient with stage IV B-NHL and CNS disease at presentation was subsequently diagnosed as having Wiskott–Aldrich syndrome and was withdrawn from the study after receiving the first two blocks of treatment. Two patients with B-ALL and CNS disease at presentation never achieved CR and died 4 and 11 months after diagnosis. Ten patients (16%) relapsed after achieving CR 4–11 months after diagnosis (median 6.4 months). Sites of relapse were CNS ($n$ = 4), bone marrow ($n$ = 2), combined CNS and bone marrow ($n$ = 3) and jaw ($n$ = 1). In six of these patients, the early treatment (block 2 and/or 3) had to be delayed or modified, and in another patient the later blocks had to be delayed because of poor clinical condition or other associated complications, mainly infectious or metabolic. Similar delays occurred in 21 patients who did not relapse and in four patients who died of toxicity. Eight of the patients who relapsed received palliative chemotherapy. Two were treated with high-dose cyclophosphamide and total-body irradiation (TBI) followed by allogeneic bone marrow transplantation (BMT). Both relapsed in the bone marrow 3 and 24 months after BMT. All have died.

One patient with CNS disease at presentation received craniospinal irradiation relapsed in the CNS and died 2 months later, whereas the remaining 12 patients remain in CR. Twenty-two patients with CNS disease at diagnosis did not receive irradiation. Seventeen are alive in CR and five relapsed and died (Table 2).

**Figure 2** Overall survival of all patients.

**Figure 3** Event-free survival of all patients

### Toxicity

Seven patients (11%) died of toxicity between 11 days and 4 months (median 1.3 months) after diagnosis. The cause of death was sepsis, bacterial or fungal ($n$ = 5), or sepsis combined with renal failure ($n$ = 2) (Table 3). Five of these patients presented with renal failure at diagnosis or after the initiation of induction therapy and all required dialysis (peritoneal or haemodialysis). Among the 43 survivors, five patients required dialysis for renal failure at presentation or shortly after the initiation of chemotherapy.

### Survival

With a median follow-up of 3.1 years from diagnosis (range 9 months to 6.3 years), 43 patients (69%) survive in complete remission (CI 55–79%) (Figure 2) with event-free survival (EFS) of

© Cancer Research Campaign 1998

**Figure 4** Overall survival of patients with B-ALL vs stage IV B-NHL

**Figure 5** Event-free survival of all patients with B-ALL vs stage IV B-NHL

69% (CI 57–79%) (Figure 3). Twenty-three patients (64%) (CI 46–78%) with B-ALL and 20 patients (74%) (CI 55–87%) with stage IV B-NHL survive (Figure 4), with EFS 66% (CI 49–79%) and 74% (CI 55–87%) respectively (Figure 5). Fifteen patients out of 24 with B-ALL and no CNS disease at diagnosis are relapse free, EFS 63% (CI 43–79%). Eight out of 11 patients with B-ALL and CNS involvement are relapse free and well, EFS 73% (CI 43–90%). Two of the long-term survivors developed mild cardiomyopathy requiring continuous captopril but remain symptom free. Two patients have features of upper motor neurone lesion in the lower limbs secondary to spinal cord compression at presentation. One patient had delayed puberty and required testosterone (Sustanon) therapy and another patient continued to have mild thrombocytopenia requiring no therapy. Another patient was diagnosed as having osteochondritis of the L1-L5 vertebrae causing backache and requiring regular analgesia. Another survivor was subsequently found to be HIV positive 4 years after the initial diagnosis of lymphoma.

### DISCUSSION

The use of a short, intensive multiagent regimen (UKCCSG MACHO protocol) resulted in improved prognosis for B-ALL patients and those with stage IV disease (Hann et al, 1988). The LMB 86 protocol improved the disease-free survival further by increasing the dose of high-dose methotrexate and cytarabine and the frequency of intrathecal medications (Rubie et al, 1988). The significance of partial substitution of ifosfamide for cyclophosphamide in the German study is difficult to interpret, but has not significantly improved the results of treatment (Reiter et al, 1989, 1995).

The role of radiotherapy to achieve local control in patients with CNS disease at presentation remains controversial. Avoidance of cranial irradiation would help to reduce long-term endocrine and neurological sequelae and is therefore desirable. The omission of cranial irradiation from the treatment of patients with advanced B-NHL and B-ALL without CNS disease at presentation did not affect the event-free survival or the risk of CNS relapse (Patte et al, 1986). In the LMB 89, the use of high-dose systemic chemotherapy resulted in event-free survival of 87% in patients with B-ALL without CNS involvement and 81% in those with CNS disease at presentation (Patte et al, 1996). Only those with CNS disease received cranial irradiation. Bowman et al (1996) reported their results of 133 patients, 74 with B-ALL and 59 with stage IV B-NHL, using intensive short-course chemotherapy regimen with intrathecal MTX and Ara-C. The 4-year EFS was 65% and 79% for B-ALL and stage IV B-NHL respectively. For those with CNS involvement, the 4-year EFS was 64% without using radiotherapy. It is of interest that in the present study CNS irradiation was selectively omitted in many patients without any apparent adverse effect on outcome.

Reduction in the total duration of therapy from 7 months to 4 months in the LMB 84 study did not lead to a difference in event-free survival and overall survival between both arms (Patte et al, 1991), but the toxic death rate was reduced from 10% to 6% in the 4-month therapy arm. It was concluded from this and another study (Schwenn et al, 1991) that long term CR can be achieved without reducing the overall survival rate by reducing the overall duration of treatment. It seems likely that a shorter regimen than 9003 could be equally effective. How high the dose of individual chemotherapy agents, e.g. cyclophosphamide, need to be is debatable.

In 1991 the Children's Cancer Study Group (CCSG) compared the results of treatment of children with high-risk B-NHL with bone marrow or CNS involvement in a randomized study using the LMB-89 and CCSG hybrid labelled 'orange'. The 2-year event-free survival was not significantly different – 80% with the LMB regimen and 84% with the CCSG regimen, but with more significant toxicity and longer hospitalization with the former (Cairo et al, 1996). The Paediatric Oncology Group (POG) 86 study used fractionated cyclophosphamide with doxorubicin and vincristine followed by MTX 1 g $m^{-2}$ and high-dose Ara-C. Eighty-one patients with B-ALL and stage IV B-NHL were treated with an event-free survival of 61% and 71% respectively. In patients with CNS disease at presentation ($n$ = 24), the event-free survival was 52% without using irradiation (Brecher et al, 1992). To compare with published studies and if the Murphy staging system is used in the present study, all patients except two who had extensive

© Cancer Research Campaign 1998

paraspinal disease would be defined as B-ALL with CNS disease. Their overall EFS is 69%.

With the 9003 regimen, despite the encouraging overall survival, early toxic death and relapse remain important causes of treatment failure. Many of these patients are extremely ill and malnourished at presentation. Toxic death shortly after diagnosis or after initial treatment is an important cause of treatment failure. Early metabolic complications (hyperuricaemia and renal failure) may precede therapy and contribute not only to early toxic death but also to subsequent intolerance of chemotherapy. Despite initiation of hyperhydration and allopurinol, toxic deaths continue to occur. The introduction of urate oxidase (uricozyme), which converts uric acid to the stable allantoic acid, may help to reduce the risk of metabolic complications (Masera et al, 1982).

Early response to induction treatment is an important prognostic factor (Patte et al, 1986; Patte et al, 1991), and delay in initiating treatment or modification of early treatment because of poor general condition may contribute to subsequent treatment failure. In this study, there was, however, no evidence that this contributed to subsequent relapse. Long-term endocrine problems were uncommon. Two patients developed mild cardiomyopathy requiring regular medication. Both are well and their cardiac dysfunction does not interfere with their daily activities.

An international study started in April 1996 is being conducted by SFOP, CCCG and UKCCSG. Children with advanced B-NHL/leukaemia are randomized to receive treatment of differing intensity. The primary aim of the study is to confirm that the event-free survival is not substantially altered by reducing the intensity and the duration of treatment. Children with CNS involvement will receive high-dose systemic chemotherapy and regular intrathecal medications but no cranial irradiation. The hope is to reduce the long-term toxicity, particularly cardiotoxicity, impaired fertility and secondary malignancy, without jeopardizing the overall success rate.

### ACKNOWLEDGEMENT

The authors thank Jane Neil for her secretarial help.

### REFERENCES

Al-Attar A, Al-Mondhiry H, Al-Bahrani Z and Al-Saleem T (1979) Burkitt's lymphoma in Iraq. Clinical study of forty-seven patients. *Int J Cancer* **23**: 14

Al-Attar A, Pritchard J, Al-Saleem T, Al-Naimi M, Alash N and Atra A (1986) Intensive chemotherapy for non-localised Burkitt's lymphoma. *Arch Dis Child* **61**: 1013

Al-Attar A, Atra A, Al-Bagdadi R, Al-Naimi M, Al-Saleem T and Pritchard J (1989) 'Debulking' surgery is unnecessary in advanced abdominal Burkitt lymphoma in Iraq. *Br J Cancer* **59**: 610–612

Allegretta GJ, Weismann SJ and Altman AJ (1985) Metabolic and space-occupying consequences of cancer and cancer treatment. *Ped Clin N Am* **32**: 601

Bowman WP, Shuster JJ, Cook B, Griffin T, Behm F, Pullen J, Link M, Head D, Carroll A, Berard C and Murphy S (1996) Improved survival for children with B-cell acute lymphoblastic leukemia and stage IV small noncleaved-cell lymphoma: a pediatric oncology group study. *J Clin Oncol* **14**: 1252–1261

Brecher M, Murphy SB, Bowman P, Sulllivan MP, Shuster J and Berard C (1992) Results of Pediatric Oncology Group (POG) 8617 (abstract 1167). *Proc Am Soc Clin Oncol* **1**: 340

Cairo M, Krailo M, Morse M, Hutchinson R, Harris R, Kjeldsberg C, Kadin M, Radel E, Steinherz L and Meadows A (1996) Disseminated non-lymphoblastic non-Hodgkin's lymphoma (DNLNHL) of childhood: a randomised phase II trial of short intensive treatment (abstract 093). *Ann Oncol* **7**: 29

Chilcote R, Krailo M, Kjeldsberg C, Kadin M, Steinherz P, Coccia P, Morse M, Reaman G and Hammond G (1991) Daunomycin plus COMP vs. COMP therapy in childhood non-lymphoblastic lymphomas. *Proc ASCO* **10**: 289

Frappaz D, Bouffet E, Biron P, Ladjaj Y, Philip I, Favrot M, Freycon F, Philippe N, Souillet G, Philip T and Brunat Mentigny M (1988) Acute surgical complications of advanced abdominal Burkitt's lymphoma in 63 children. In *Meeting of the International Society of Paediatric Oncology*, Trondheim, Abstract 139.

Gentet JC, Patte C, Quintana E, Bergeron C, Rubie H, Pein F, Demaille MC, Philip T and Raybaud C (1990) Phase II study of cytarabine and etoposide in children with refractory or relapsed non-Hodgkin's lymphoma: a study of the French Society of Pediatric Oncology. *J Clin Oncol* **8**: 661–665

Hann IM, Eden OB, Barnes J and Pinkerton CR (1988) MACHO chemotherapy for Stage IV B cell lymphoma and a cell acute lymphoblastic leukemia of childhood. *Br J Haem* **76**: 359–364

Lynch RE, Kjellstrand CM and Coccia PF (1977) Renal and metabolic complications of childhood non-Hodgkin's lymphoma. *Semi Oncol* **4**: 235

Masera G, Jankovic M, Zurlo MG, Locasciulli A, Rossi MR, Uderzo C and Recchia M (1982) Urate-oxidase prophylaxis of uric acid-induced renal damage in Childhood leukemia. *J Ped* **100**: 152–155

Patte C, Philip T, Rodary C, Bernard A, Zucker JM, Bernard JL, Robert A, Rialland X, Benz-Lemoine E and Demeocq F (1986) Improved survival rate in children with stage III and IV B cell non-Hodgkin's lymphoma and leukemia using multi-agent chemotherapy: results of a study of 114 children from the French Pediatric Oncology Society. *J Clin Oncol* **4**: 1219–1226

Patte C, Leverger G, Perel Y, Rubie H, Otten J, Nelken B, Gentet JC, Lumley L De, Berendt H, Brugleres L, for the SFOP (1990). Updated results of the LMB 86 protocol of the French Pediatric Oncology Society (SFOP) for B-cell non-Hodgkin's lymphomas 9B-NHL) with CNS involvement (CNS+) and B-ALL (abstract 22). *Med Ped Oncol* **18**: 397

Patte C, Philip T, Rodary C, Zucker JM, Behrendt H, Gentet JC, Lamagnère JB, Otten J, Dufillot D, Pein F, Caillou B and Lemerle J (1991) High survival rate in advanced stage B cell lymphoma and leukemias without CNS involvement with a short intensive polychemotherapy: results from the French Pediatric Oncology Society of a randomised trial of 216 children. *J Clin Oncol* **9**: 123–132

Patte C, Michon J, Behrendt H, Leverger G, Frappaz D, Robert A, Mechinaud F, Bertrand Y, Perel C, Coze C and Nelken B (1996) Updated results of the LMB 89 protocol of the SFOP (French Pediatric Oncology Society) for childhood B-cell lymphoma and leukemia (ALL) (abstract 96). *Ann Oncol* **7**: 30

Philip T, Lenior GM, Bryon PA, Gerard-Marchant R, Souillet G, Philippe N, Freycon F and Brunat-Mentigny M (1982) Burkittt type lymphoma in France among non-Hodgkin malignant lymphomas in caucasian children. *Br J Cancer* **45**: 670–678

Reiter A, Sauter S, Kabisch H, Ritter J, Harbot J, Gadner H and Riehm H (1989) Probability for cure as related to therapy in childhood B-type acute lymphoblastic leukemia (B-ALL) in three consecutive BFM trials. *Med Ped Oncol* **17**: 321

Reiter A, Schrappe M, Parwaresch R, Henze G, Muller-Weihrich S, Sauter S, Sykora KW, Ludwig WD, Gadner H and Riehm H (1995) Non-Hodgkin's lymphomas of childhood and adolescence: results of a treatment stratified from biological subtypes and stage – a report of the Berlin-Frankfurt-Munster Group. *J Clin Oncol* **13**: 359–372

Rubie H, Patte C, Leverger G, Philip T, Quintana E, Rialand X, Boutard P, Benz-Lemoine E, Otten J, Taboureau O, Frappaz D, for the SFOP (1988) Preliminary results of the protocol LMB 86 of the French Pediatric Oncology Society for B cell non-Hodgkin's lymphoma with CNS involvement and B-ALL In *20th Meeting of the International Society of Paediatric Oncology*, Trondheim, Abstract 84

Schwenn MR, Blattner SR, Lynch E and Weinstein HJ (1991) HiC-COM: A 2-month intensive chemotherapy regimen for children with stage III and IV Burkitt's lymphoma and B-cell acute lymphoblastic leukemia. *J Clin Oncol* **9**: 133–138

© Cancer Research Campaign 1998